# Does mass management of chronic hepatitis C protect the Egyptian population against fulminant coronavirus disease-2019? “Postulating a hypothesis”

**DOI:** 10.1186/s43168-022-00120-2

**Published:** 2022-03-29

**Authors:** Essamedin M. Negm

**Affiliations:** Anesthesia, Intensive Care and Pain Management Department, Zagazig University, Zagazig, Egypt

**Keywords:** COVID-19, Hepatitis C, DAAs, RAS, NK cells, Coagulopathy, Prophylaxis

## Abstract

Coronavirus disease (COVID-19) is caused by the pathogenic virus severe acute respiratory syndrome coronavirus 2 (SARS-CoV-2). Egypt has launched a national treatment program to provide a cure for Egyptian patients infected with hepatitis C virus (HCV). A common mechanism is shared between both the anticipated and unexpected aspects of COVID-19. The activity of the renin-angiotensin system (RAS) is intrinsically high in the lungs, which is a major source of ACE and hence a significant site of systemic synthesis of Ang II. Angiotensin-converting enzyme 2 (ACE2) is the cellular receptor for SARS-CoV-2, the etiological agent of the COVID-19 disease. ACE-2 and its angiotensin 1–7 (Ang 1–7) product, which acts on the Mas oncogene receptor, have been shown to play a protective role in fibrogenesis and inflammation of many organs, including the liver and lung. Antiviral treatment with interferon (IFN) in conjunction with ribavirin in patients with chronic hepatitis C reduces serum ACE activity and indirectly affects liver parenchyma fibrogenesis. The antifibrotic activity of sofosbuvir plus daclatasvir (SOF/DAC) is independent of its antiviral action. Elimination of HCV infection by DAA therapy in patients with chronic hepatitis C could improve natural killer (NK) activity by increasing the frequency of CD 16+ CD 56+ NK cells. COVID-19 individuals exhibit enhanced platelet activation and aggregation, as well as platelet-monocyte aggregation, which is linked to coagulative disorders. Lower systemic inflammation and enhanced hepatic synthesis of both pro- and anti-coagulant factors were noticed soon after antiviral therapy. In order to protect against the severity of COVID-19, treatment of chronic hepatitis C has been observed as a possible key as a prophylaxis beside the vaccine and should be tested for evidence or rejection of observation.

## Introduction

Many intensivists (consultants and heads of COVID-19 isolation ICUs) interested in COVID-19 research and management observed during 1st and 2nd wave of epidemic that while chronic hepatitis C virus (HCV) is endemic in Egypt and WHO reported that it is one of the risk factors for severe COVID 19, the admission of these populations is very low and of low severity in ICUs even they have well-known risk factors like hypertension, diabetes, obesity, malignancy, and smoking and many healthcare workers who were previously treated with hepatitis C antivirals recorded their exposure to COVID-19 patients at their infectivity period without catching the disease and in the following sequences, the summarized scientific context to find the explanation has been established; particularly, we found that those who received direct antiviral treatment for treatment of hepatitis C look like immunized against COVID-19. I hypothesize that Modulation of the RAS by the chronic hepatitis C virus treatment either by (Daclatasvir and Sofosbuvir), (Daclatasvir and Sofosbuvir plus Ribavirin), or (Ribavirin and Interferon) protocols together with restoration of NK cell effector functions and lowering systemic inflammation and enhanced hepatic synthesis of both pro- and anti-coagulant factors could give an explanation to the low incidence of COVID-19 among Egyptian population and can help in the management of the epidemic and will help Egypt’s herd immunity to grow more quickly. In addition, it may play a role in COVID 19 prophylaxis.

## Evidence synthesis for the hypothesis

The coronavirus disease-2019 (COVID-19) is not a simple viral pneumonia with the numerous unusual pathophysiological, biochemical, and hematological effects having been described. Of particular interest is the interaction of severe acute respiratory syndrome coronavirus-2 (SARS-CoV-2) and the renin-angiotensin system (RAS) via angiotensin-converting enzyme-2 (ACE-2), the receptor used by the SARS-CoV-2 to gain access to cells. This interaction could trigger an explanation for the several unusual clinical findings observed in COVID-19 [1]. A common mechanism is shared between both the anticipated and unexpected aspects of COVID-19: silent hypoxia, atypical acute respiratory distress syndrome (ARDS), stroke, loss of smell, myocarditis, and elevated mortality in the elderly, in men, in African Americans, and in obesity, diabetes, and cancer patients. All bear the fingerprints of the imbalance of the renin-angiotensin system (RAS), indicating that the common culprit is RAS [2]. We also add our daily and continuous observation and explanation as regards the role of anti-hepatitis C medications to protect hepatic patients to this theory.

**Fig. 1:**
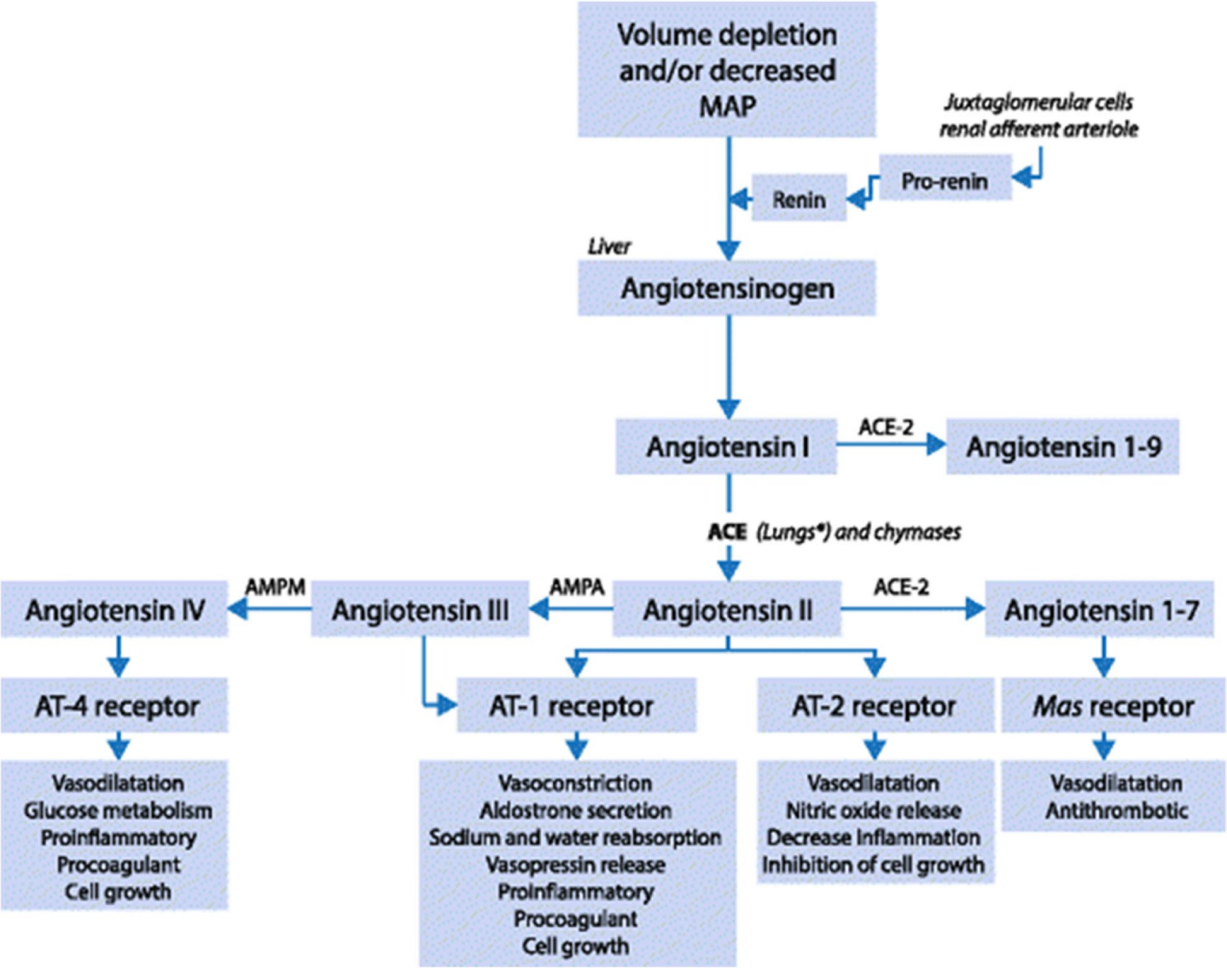
Overview of the renin-angiotensin system. MAP, mean arterial blood pressure; AT, angiotensin; ACE, angiotensin-converting enzyme; AMPA, aminopeptidase A; AMPM, aminopeptidase M; *, ACE is present mainly in lung capillaries, although it can also be found in the plasma and vascular beds of other organs, such as the kidneys, brain, heart, and skeletal muscle

The RAS (Fig. 1) [3] comprises a carefully balanced and controlled cascade of hormones and receptors involving multiple organ systems. Angiotensinogen, continuously released from the liver into the circulation, is cleaved to angiotensin I (AngI) by renin which is released from the kidney. AngI catalyzed by angiotensin-converting enzyme (ACE) located on vascular endothelium, and most abundantly found in lung tissue to produce the active end product angiotensin II (AngII). AngII acts mainly via angiotensin 1 (AT1) receptors. The activation of this AngII/AT1 classic pathway leads to a number of unfavorable effects. It results not only in the well-known vasoconstrictive effects, but also a host of potentially detrimental effects on the endothelium, inflammation, vascular permeability facilitating pulmonary edema, and coagulation [4–7].

There is also a protective RAS pathway as AngI is converted to Ang (1–9) and AngII converted to Ang (1–7) by ACE-2. Ang (1–7) primarily exerts effects on the MAS receptors causing vasodilation, natriuresis, diuresis, anti-proliferative, and anti-inflammatory effects. This pathway is the so-called RAS protective pathway [5, 6] which may be suppressed in COVID-19 leading to overstimulation of the classic pathway with adverse cardiovascular and respiratory effects, hypercoagulation, endothelial dysfunction, inflammation, and insulin resistance [1].

The AngII/AT1 axis of the RAS system plays role in the development of fibrotic diseases, while the other counter components, ACE-2/Ang1–7/Mas axis, have shown potential protective effects in organ fibrosis [1, 8]. Thus, stimulating this protective arm of RAS would be a prophylactic or treatment option for liver and lung fibrosis.

In both animal and human studies, angiotensin-converting enzyme inhibitors and angiotensin receptor blockers slow the progression of fibrosis. In a bile duct ligation mouse model, ACE-2 supplementation prevents liver fibrosis [9].

Chronic HCV Interferon-associated antiviral therapy (IFN) with ribavirin has been reported to decrease serum ACE activity and indirectly affect liver parenchyma fibrogenesis. Ribavirin also exerts an immunomodulatory action of the host to the virus by shifting a Th2 response in favor of a Th1 phenotype. Th2 response and production of type 2 cytokines such as IL-4, IL-5, and IL-10 stimulate the humoral response which enhances immunity toward the virus [10]. In addition, their sustained virologic response (SVR) was shown in a study by Marcellin and colleagues with a mean follow-up of 4 years, showing 96% undetectable serum HCV RNA [11]. So, it can act as a prophylaxis in our epidemic.

Ribavirin also demonstrated an inhibitory effect on the enzyme TMPRSS2. TMPRSS2 is a membrane protease for the priming of ACE2, which is a critical step for SARS-CoV-2 fusion [12].

Highly effective direct-acting antivirals (DAAs)—daclatasvir and sofosbuvir (SOF/DAC)—have recently been used for HCV elimination. The antifibrotic activity of SOF/DAC is independent of its antiviral action. The molecular events associated with this effect were the downregulation of tumor necrosis factor alpha/nuclear factor kappa-light-chain-enhancer of activated B cells (TNF-α/NF-κB) signaling pathway and induction of B-cell lymphoma 2 (Bcl-2) [13]. Observational clinical data support the potential of anti-TNF therapies as a treatment for COVID-19. Data from the SECURE-IBD registry suggest that when patients with inflammatory bowel disease develop COVID-19, those on anti-TNF therapies do just as well and possibly better than those on alternative agents. It was found that anti-TNF therapy was inversely related to the composite outcome of death or hospital admission for COVID-19. A functional renin/angiotensin system (RAS) in the target cell is necessary for the induction of alveolar epithelial cells (AEC) apoptosis by TNF-alpha. TNF-alpha exposure increased Ang II concentration [14, 15].

DAAs for HCV infection have resulted in high rates of sustained virologic response (SVR) following 8 to 24 weeks of treatment. In the IMPACT study, virologic response to simeprevir, sofosbuvir, and daclatasvir was durable over 3 years and all patients who reached the 3-year follow-up time point maintained SVR [16].

Another factor which strengthens the protection is the relation between management of hepatitis C and natural killer (NK) cells. In patients with chronic hepatitis C, eliminating HCV infection with DAA therapy could increase NK activity by increasing the frequency of NK cells and of CD 16+ CD 56+ NK cells [17]. NK cells are a type of innate immune cell that acts as a first line of defense against acute infections and cancer, as well as controlling the adaptive immune response [18]. Natural killer cells play a central role in maintaining immune homeostasis, which is crucial when facing the challenge of a novel pathogen. Preliminary studies in COVID-19 patients with serious disease suggest a reduction in NK cell number and function leading to a decrease in infected and activated cell clearance and unchecked increase in tissue-damaging inflammation. Restoration of NK cell effector functions has the potential to correct the delicate immune balance needed for successful SARS-CoV-2 infection clearance [19]. Furthermore, according to Bao et al., natural killer cells can be used as a universal COVID-19 treatment [20].

When SARSCoV-2 infects endothelial cells via ACE2, this receptor is internalized and downregulated, preventing it from functioning normally. The buildup of angiotensin II would result in an increase in prothrombotic signaling as a result of this downregulation (Ang II) [21]. SARS-CoV-2 interacts with platelets and megakaryocytes via an ACE2-independent mechanism, and this interaction may influence alternative receptor expression linked to COVID-19 coagulation dysfunction [22]. COVID-19 individuals exhibit enhanced platelet activation and aggregation, as well as platelet-monocyte aggregation, which is linked to coagulative disorders [23], which emphasizes platelets’ crucial role in SARS-CoV-2 infection and immunopathology [24]. Alterations of primary hemostasis (platelet adhesion, activation, and aggregation) have been liked to chronic HCV-induced liver cirrhosis, with platelet hypofunction being more noticeable in the early stages of the disease compared to the later ones [25].

The effect of DAAs on coagulopathy in patients with HCV-related cirrhosis was investigated in a prospective study utilizing thrombomodulin-modified thrombin generation test; SVR is associated with a significant decrease of in vitro hypercoagulability. This effect, which was likely due to lower systemic inflammation and enhanced hepatic synthesis of both pro- and anti-coagulant factors, was noticed soon after antiviral therapy ended and lasted for up to a year [26]. Patients who do not have their hypercoagulability reversed following DAAs are at a higher risk of problems [27].

Egypt has the highest prevalence of hepatitis C virus (HCV) worldwide, with a prevalence rate of 21.9% in 1995–1996 among adults [28]. Antibody prevalence among adults aged 15–59 years was 14.7% in 2009 and 10.0% in 2015, according to the Egyptian Demographic and Health Surveys (EDHS) [29].

Since 2007, care for HCV in Egypt has been one of the top national priorities. Egypt has launched a national treatment program intending to provide cure for Egyptian HCV-infected patients. Mass HCV treatment program had started using pegylated interferon and ribavirin between 2007 and 2014. In May 2016, the World Health Assembly set targets for the elimination of viral hepatitis, including reaching 90% diagnosis, 80% treatment coverage, and a 65% reduction in related mortality by 2030 [30]. The Egyptian National Committee for the Control of Viral Hepatitis did its best to provide DAAs to Egyptian HCV patients [31]. Planning started in May 2018 with the goals of the Ministry of Health were to screen everyone 18 years of age or older in Egypt with a target population of 62.5 million within 1 year and to provide treatment to all those with HCV viremia. All patients were treated with sofosbuvir (400 mg daily) plus daclatasvir (60 mg daily) with or without ribavirin for duration of 12 or 24 weeks, depending on the presence or absence of cirrhosis and the stage of cirrhosis. Of the target population, 49,630,319 persons (79.4%) participated spontaneously in the screening between October 1, 2018, and April 30, 2019 [32].

Between 2010 and 2014, the number of confirmed cases of acute viral hepatitis C infection in the USA rose by more than 2.5 times. According to recent epidemiologic surveys, at least 4.6 million people have tested positive for HCV antibodies, and around 3.5 million are currently infected with the virus [33]. HCV infection is now the most common blood-borne viral infection in the USA, killing more people than HIV and being the primary cause of cirrhosis, liver cancer, liver failure, and liver-related deaths [34].

Although HCV is one of the risk factors listed by WHO for severe COVID-19, existing literature of a case series of 5700 COVID-19 patients in multiple hospitals in the New York City area identified only <0.1% of these patients have infections [35].

Nassar et al. studied the characteristics, outcomes, and risk factors for inhospital death of critically ill patients with COVID-19 pneumonia admitted to Cairo University Hospitals in Egypt, where chronic liver disease patients had the lowest incidence of all comorbidities, with zero mortality [36]. A retrospective study identifying risk factors of patients requiring ICU admission during hospital stay was collected from Ain Shams University Isolation-Hospital which records low incidence of ward admission with Zero ICU admission [37]. Other supportive findings were also found in several Egyptian centers [38–40]. A study by Negm et al. (NCT04757272) with unpublished results (pending publishing) on hospitalized critically ill 1053 COVID-19 patients supported the explanation of the current hypothesis, where patients treated with DAAs had well-documented favorable outcomes between chronic hepatitis C patients.

Pre-existing metabolic cirrhosis appears to be associated with greater mortality among COVID-19 patients, whereas HCV antibodies in hepatitis C patients may indicate “safety” against COVID-19 was a trial of explanation by a previous study [41]. We totally agree with the observation but not with the explanation as just infection is not a protection and even actually may be one of the risk factors for severity as in vivo platelet activation is thought to be caused directly by HCV-induced inflammatory and immunological processes in the liver tissues [42].

These observations and explanations were the initiators to perform an investigation hypothesizing that modulation of RAS by the chronic hepatitis C virus treatment either by daclatasvir and sofosbuvir, daclatasvir and sofosbuvir plus ribavirin, or ribavirin and interferon protocols which usually followed by hepatitis B vaccine with the restoration of natural killer(NK) cells plays together with coagulation balance a role in COVID-19 prophylaxis and may give the protection against severe COVID-19 among these populations in Egypt and may play a role beside vaccination for better eradication of the epidemic.

## Conclusions

The low incidence and severity of COVID-19 among chronic liver disease patients admitted to Egyptian hospitals might be explained by the mass management of chronic hepatitis C in Egypt. Understanding the mechanisms may help in better characterization of the disease and investigating possible therapeutic options. Intervention studies are needed to prove the effectiveness of DAAs as a prophylaxis for COVID 19. We will see if our findings will help Egypt’s herd immunity grow more quickly together with the current vaccination.

### Statement of significance and recommendations

Modulation of RAS by the chronic hepatitis C virus treatment either by daclatasvir and sofosbuvir, daclatasvir and sofosbuvir plus ribavirin, or ribavirin and interferon protocols which usually followed by hepatitis B vaccine with restoration of natural killer (NK) cells plays together with coagulation balance a role in COVID-19 prophylaxis and may give the protection against severe COVID-19.

## Data Availability

Not applicable.

## Abbreviations

COVID-19: Coronavirus disease-2019
SARS-CoV-2: Severe acute respiratory syndrome coronavirus 2
RAS: Renin-angiotensin system
ACE II: Angiotensin-converting enzyme II
Ang II: Angiotensin II
NK cells: Natural killer cells
SOF/DAC: Sofosbuvir plus daclatasvir
ICUs: Intensive care units
WHO: World Health Organization
ARDS: Acute respiratory distress syndrome
AT1: Angiotensin 1 receptors
IFN: Interferon
IL: Interleukin
DAA: Direct-acting antiviral
TNF: Tumor necrosis factor alpha
Bcl: B-cell lymphoma
AEC: Alveolar epithelial cells
SVR: Sustained virologic response
HIV: Human immunodeficiency virus
HCV: Hepatitis C virus

## References

[1] Wiesea OJ, Allwoodb BW, Zemlina AE (2020). COVID-19 and the renin-angiotensin system (RAS): a spark that sets the forest alight?. Med Hypotheses.

[2] Czick M, Shapter C, Shapter R (2020). COVID’s razor: RAS imbalance, the common denominator across disparate, unexpected aspects of 10 of 13 - CLARK COVID-19. Diabetes Metab Syndr Obes.

[3] Corrêa TD, Takala J, Jakob SM (2015). Angiotensin II in septic shock. Crit Care.

[4] Yang T, Xu C (2017). Physiology and pathophysiology of the intrarenal renin-angiotensin system: an update. J Am Soc Nephrol.

[5] Paz Ocaranza M, Riquelme JA, Garcia L, Jalil JE, Chiong M, Santos RAS (2020). Counter-regulatory renin-angiotensin system in cardiovascular disease. Nat Rev Cardiol.

[6] Dhanachandra SK, Karnik SS (2017) Angiotensin receptors: structure, function, signaling and clinical applications. J Cell Signal 01(02) Available from: https://pubmed.ncbi.nlm.nih.gov/27512731/10.4172/jcs.1000111 PMC4976824 27512731

[7] Imai Y, Kuba K, Rao S, Huan Y, Guo F, Guan B (2005). Angiotensin-converting enzyme 2 protects from severe acute lung failure. Nature.

[8] Li W, Moore MJ, Vasllieva N, Sui J, Wong SK, Berne MA (2003). Angiotensin-converting enzyme 2 is a functional receptor for the SARS coronavirus. Nature.

[9] Mak KY, Chin R, Cunningham SC (2015). ACE2 therapy using adeno-associated viral vector inhibits liver fibrosis in mice. Mol Ther.

[10] Te HS, Randall G, Jensen DM (2007). Mechanism of action of ribavirin in the treatment of chronic hepatitis C. Gastroenterol Hepatol (N Y).

[11] Marcellin P, Boyer N, Gervais A, Martinol M, Pouteau M, Castelnau C (1997). Long-term histologic improvement and loss of detectable intrahepatic HCV RNA in patients with chronic hepatitis C and sustained response to interferon-α therapy. Ann Intern Med.

[12] Mehmet Altay Unal, Ceylan Verda Bitirim, Gokce Yagmur Summak, Sidar Bereketoglu, Inci Cevher Zeytin, Omur Bul, et al (2020). Ribavirin shows antiviral activity against SARS-CoV-2 and downregulates the activity of TMPRSS2 and the expression of ACE2 In Vitro. 10.1101/2020.12.04.410092. 10.1139/cjpp-2020-0734 33689451

[13] Zakaria S, El-Sisi AE (2020). Daclatasvir and sofosbuvir mitigate hepatic fibrosis through downregulation of TNF-α / NF-κB signaling pathway. Curr Mol Pharmacol.

[14] Brenner EJ, Ungaro RC, Gearry RB, Kaplan GG, Kissous-Hunt M, Lewis JD (2020). Corticosteroids, but not TNF antagonists, are associated with adverse COVID-19 outcomes in patients with inflammatory bowel diseases: results from an international registry. Gastroenterology.

[15] Wang R, Alam G, Zagariya A, Gidea C, Pinillos H, Lalude O, Choudhary G, Oezatalay D, Uhal BD (2000). Apoptosis of lung epithelial cells in response to TNF-alpha requires angiotensin II generation de novo. J Cell Physiol.

[16] Lawitz E, Mangia A, Wyles D, Rodriguez-Torres M, Hassanein T, Gordon SC (2013). Sofosbuvir for previously untreated chronic hepatitis C virus infection. N Engl J Med.

[17] Moustafaa HM, Abdel-Gawada M, Salaheldinb EM, Ibrahimc NFS (2021). Restoration of natural killer cell activity in chronic hepatitis Cinfected Egyptian patients treated by sofosbuvir/daclatasvir with or without ribavirin. Al-Azhar Assiut Med J.

[18] Schuster IS, Coudert JD, Andoniou CE, Degli-Esposti MA (2016). “Natural regulators”: NK cells as modulators of T cell immunity. Front Immunol.

[19] van Eeden C, Khan L, Osman MS, Tervaert JWC (2020). Natural killer cell dysfunction and its role in COVID-19. Int J Mol Sci.

[20] Bao C, Tao X, Cui W, Hao Y, Zheng S, Yi B (2021). Natural killer cells associated with SARS-CoV-2 viral RNA shedding, antibody response and mortality in COVID-19 patients. Exp Hematol Oncol.

[21] Gonzalez-Porras JR, Belhassen-Garcia M, Lopez-Bernus A (2020). Low molecular weight heparin in adults inpatient COVID-19.

[22] Shen S, Zhang J, Fang Y, Lu S, Wu J, Zheng X, Deng F (2021). SARS-CoV-2 interacts with platelets and megakaryocytes via ACE2-independent mechanism. J Hematol Oncol.

[23] Hottz ED, Azevedo-Quintanilha IG, Palhinha L, Teixeira L, Barreto EA, Pão CRR (2020). Platelet activation and platelet-monocyte aggregates formation trigger tissue factor expression in patients with severe COVID-19. Blood..

[24] Koupenova M, Freedman JE (2020). Platelets and COVID-19: inflammation, hyperactivation and additionalquestions. Circ Res.

[25] Ghozlan MF, Saad AA, Eissa DS, Abdella HM (2013). Evaluation of platelet dysfunction in viral liver cirrhosis ( relationship to disease severity). Egypt J Haematol.

[26] Russo FP, Zanetto A, Campello E, Bulato C, Shalaby S, Spiezia L (2018). Reversal of hypercoagulability in patients with HCV-related cirrhosis after treatment with direct-acting antivirals. Liver Int.

[27] Zanetto A, Simioni P, Russo FP (2021). Hepatic benefits of HCV cure: don’t forget coagulation!. J Hepatol.

[28] Guerra J, Garenne M, Mohamed MK, Fontanet A (2012). HCV burden of infection in Egypt: results from a nationwide survey. J Viral Hepat.

[29] Anwar WA, El Gaafary M, Girgis SA, Rafik M, Hussein WM, Sos D (2021). Hepatitis C virus infection and risk factors among patients and health-care workers of Ain Shams University hospitals, Cairo, Egypt. PLoS ONE.

[30] World Health Organization (2016) Global Health sector strategy on viral hepatitis 2016-2021: towards ending viral hepatitis. WHO Available at https://apps.who.int/iris/bitstream/handle/10665/246177/WHO-HIV-2016.06eng.pdf;jsessionid=8BBB114FF64FC1E901805FA54462BE1?sequence=1

[31] Omran D, Alboraie M, Zayed RA (2018). Towards hepatitis C virus elimination: Egyptian experience, achievements and limitations. World J Gastroenterol.

[32] El-Zanaty and Associates (2015). Egypt health issues survey 2015.

[33] Edlin BR, Eckhardt BJ, Shu MA (2015). Toward a more accurate estimate of the prevalence of hepatitis C in the United States. Hepatology..

[34] Ly KN, Xing J, Klevens RM (2012). The increasing burden of mortality from viral hepatitis in the United States between 1999 and 2007. Ann Intern Med.

[35] Richardson S, Hirsch JS, Narasimhan M, Crawford JM, McGinn T, Davidson KW (2020). Presenting characteristics, comorbidities, and outcomes among 5700 patients hospitalized with COVID-19 in the new York City area. JAMA..

[36] Nassar Y, Mokhtar A, Elhadidy A, Elsayed M, Mostafa F, Rady A (2021). Outcomes and risk factors for death in patients with coronavirus disease-2019 (COVID-19) pneumonia admitted to the intensive care units of an Egyptian University hospital. A retrospective cohort study. J Infect Public Health.

[37] Fouad SH, Allam MF, Ibrahim S, Okba AA, Roman SW, Hosny A (2021). ICU admission of COVID-19 patients: identification of risk factors. Egypt J Anaesth.

[38] Khamiss AM, El-Dahshan M, El-Ghamery F, Aggag M, Hashim A, Eliwa A (2021). Outcomes of COVID-19 in Egyptian patients. Al-Azhar Med J.

[39] Omran D, Al Soda M, Bahbah E, Esmat G, Shousha H, Elgebaly A (2021). Predictors of severity and development of critical illness of Egyptian COVID-19 patients: a multicenter study. PLoS One.

[40] Hafez MZE, Nassar AY, Nassar GAY, Abdel Hafez NF, Hamed HA, Saleem TH (2021). Epidemiology of coronavirus disease (COVID-19) in Assiut Province in Egypt. AJRID.

[41] Mangia A, Cenderello G, Verucchi G, Ciancio A, Fontana A, Piazzolla V, Minerva N, Squillante MM, Copetti M (2020). Is positivity for hepatitis C virus antibody predictive of lower risk of death in COVID-19 patients with cirrhosis?. World J Clin Cases.

[42] Witters P, Freson K, Verslype C, Peerlinck K, Hoylaerts M, Nevens F (2008). Review article: blood platelet number and function in chronic liver disease and cirrhosis. Aliment Pharmacol Ther.

